# The effect of motivational interviewing and/or cognitive behaviour therapy techniques on gestational weight gain – a systematic review and meta-analysis

**DOI:** 10.1186/s12889-023-15446-9

**Published:** 2023-04-01

**Authors:** Helen Nightingale, George Mnatzaganian, Leesa Hooker, Stephen Barrett, Michael Kingsley

**Affiliations:** 1 Rural Department of Nursing & Midwifery, La Trobe Rural Health School, La Trobe University, Bendigo, Australia; 2 Rural Department of Community Health, La Trobe Rural Health School, La Trobe University, Bendigo, Australia; 3 Judith Lumley Centre, School of Nursing and Midwifery, La Trobe University, Bundoora, Australia; 4 Research and Innovation, Bendigo Health, Bendigo, Australia; 5 Holsworth Research Initiative, La Trobe Rural Health School, La Trobe University, Bendigo, Australia; 6 Department of Exercise Sciences, University of Auckland, Auckland, New Zealand

**Keywords:** Gestational weight gain, Behaviour change, Motivational interviewing, Cognitive behaviour therapy, Pregnancy, Systematic review

## Abstract

**Background:**

Women with gestational weight gain (GWG) that is below or above recommendations are at risk of adverse perinatal outcomes. Motivational interviewing and/or cognitive behaviour therapy have demonstrated efficacy in initiating and sustaining behaviour change, including weight control. The objective of this review was to investigate the effect of antenatal interventions that include components of motivational interviewing and/or cognitive behaviour therapy on gestational weight gain.

**Methods:**

This review was designed and reported in accordance with guidelines outlined in the Preferred Reporting Items for Systematic Reviews and Meta-Analyses (PRISMA) statement. Five electronic databases were systematically searched to March 2022. Randomised controlled trials evaluating interventions with identified components of motivational interviewing and/or cognitive behaviour therapies were included. Pooled proportions of appropriate GWG and GWG above or below guidelines, and standardised mean difference for total gestational weight gain, were calculated. Risk of bias in included studies was evaluated using the Risk of Bias 2 tool, and the Grades of Recommendation, Assessment, Development and Evaluation (GRADE) approach was used to evaluate the quality of evidence.

**Results:**

Twenty-one studies (8030 participants) were included. Overall, MI and/or CBT interventions had a small effect on the total gestational weight gain (SMD: -0.18, 95% confidence interval: -0.27 to -0.09, *p* < 0.001) and improved the proportion of women achieving recommended gestational weight gain (29% versus 23% in the comparison, *p* < 0.001). The GRADE assessment indicated that overall quality of evidence is very uncertain, however sensitivity analyses to account for high risk of bias produced similar results to original meta-analyses. The magnitude of effect was greater in women with overweight or obesity when compared to women with BMI < 25 kg/m^2^.

**Conclusion:**

Motivational interviewing and/or cognitive behaviour therapy techniques may be effective for promoting healthy gestational weight gain. Nevertheless, a high proportion of women do not achieve recommended gestational weight gain. Future interventions should consider factors, including clinician and consumer perspectives, in the design and delivery of psychosocial interventions that aim to support healthy gestational weight gain.

**Trial registration:**

The protocol for this review was registered with the PROSPERO International register of systematic reviews (registration number CRD42020156401).

**Supplementary Information:**

The online version contains supplementary material available at 10.1186/s12889-023-15446-9.

## Background

Weight gain during pregnancy is physiologically normal and an expected response to the growth of the fetus, placenta and amniotic fluid, and changes in body composition and metabolism [1]. However, gestational weight change outside of recommended guidelines is acknowledged to influence a range of important perinatal outcomes for mothers and infants [2–4]. Gestational weight gain [GWG] guidelines have been developed by the United States-based Institute of Medicine (IoM) (Table 1) using observational data comparing GWG with perinatal outcomes [2, 3]. Although evidence suggests that GWG patterns are similar across different populations [5], these guidelines have been inconsistently adopted by other countries [6, 7].

**Table 1:** Recommended gestational weight gain stratified by body mass index category [3]

Pre-pregnancy BMI	Recommended GWG
< 18.5 kg/m^2^	12.5 – 18.0 kg
18.5 – 24.9 kg/m^2^	11.5 – 16.0 kg
25 – 29.9 kg/m^2^	7.0 – 11.5 kg
≥ 30 kg/m^2^	5.0 – 9.0 kg

*BMI* body mass index, *GWG* gestational weight gain

Weight change during pregnancy that is either above or below GWG guidelines is associated with serious short- and long-term consequences for mothers and infants [4, 8]. Specifically, GWG below these guidelines has been linked with premature birth and infants that are small for gestational age [4, 8, 9], and GWG above guidelines has been linked to maternal complications such as: gestational diabetes mellitus [GDM], hypertensive disorders of pregnancy and caesarean birth [4, 8]. Gestational weight gain above guidelines is also associated with longer-term impacts on maternal weight and body mass index [BMI] such as increased risk of maternal weight retention, future obesity, abdominal adiposity and associated risk of chronic health impacts such as insulin resistance [10–13]. Depending on pre-pregnancy BMI category, those with GWG above guidelines are 1.5 to 3 times more likely to have an 18-month postpartum weight retention above 2 kg [14], and to retain up to 4.7 kg on average after 15 years postpartum [15]. Postpartum weight retention is a contributor to future obesity, with one study finding 89% of women with a healthy pre-pregnancy BMI were overweight or obese 5 years postpartum [16]. Additionally, GWG above guidelines has been associated with increased risk for morbidities in infants, including admission to neonatal intensive care, hypoglycaemia or respiratory distress, large for gestational age [LGA], low 5-min APGAR (Appearance, Pulse, Grimace, Activity, Respiration) score, and long-term risk of offspring overweight or higher BMI [10, 12, 17, 18]. Further, GWG above guidelines increases the risk of offspring obesity by 1.4 to 1.8 times [10, 19] with increased propensity for higher levels of biomarkers of adverse cardio-metabolic health in male offspring [10]. Higher BMI and GWG above guidelines have been suggested to also alter maternal and infant gastrointestinal microbiomes, with detrimental consequences to many areas of health and disease [20, 21].

GWG outside recommendations has strong links with adverse maternal and infant health, and optimal GWG represents an opportunity to improve population health [22]. Pre-pregnancy overweight or obesity, as categorised by the World Health Organization [23], amplifies the risk of adverse perinatal outcomes associated with GWG outside recommended ranges, such as up to two-fold increase in risk for pre-eclampsia and up to three-fold increase in risk for GDM [2, 11, 24]. Furthermore, maternal obesity incurs an increased risk for a range of congenital abnormalities in the fetus [25].

Despite the likely benefits of meeting GWG guidelines, most women do not achieve recommended weight gain, with around half of women gaining weight above guidelines and 20–23% gaining below guidelines [4, 26]. Women who are overweight or obese at the beginning of pregnancy are more likely to have GWG above guidelines than women with BMI < 25 kg/m^2^ [2, 8], and the proportion of pregnant women who are overweight or obese has increased over the past two decades [27]. Current recommendations for GWG stratified by pre-pregnancy BMI are shown in Table 1, and weight change outside of these recommendations can be considered as GWG above or below these guidelines [3].

Weight change during pregnancy can be influenced by a range of complex factors, such as biological, environmental, economical, psychological and sociocultural characteristics that affect eating and activity behaviours [3, 28], and social context and attitudes towards weight in pregnancy may contribute to GWG-related health behaviours [29]. Further debate relating to GWG recommendations and interventions urges consideration of likely adverse impacts on GWG from weight stigma, systemic racism, low income and education, such as the chronic stress from these that influence physiology, metabolism and accumulation of excess body fat [30, 31]. Other authors argue that prescriptive interventions change nothing about the participant’s way of living once the intervention ceases, and need to account for individual variation in mental, emotional and living conditions [22].

Interventions to promote healthy GWG initially focused on diet and/or exercise alone, with small and inconsistent effects [32]. The most recent revision of GWG recommendations acknowledged the complex interplay between factors that influence GWG and advocated for future research to include consideration of behavioural and psychosocial determinants of GWG [3]. Behaviour change approaches such as motivational interviewing [MI] and cognitive behaviour therapy [CBT] are commonly used to address determinants of motivation, self-efficacy and self-regulation.

MI is a collaborative, guiding technique for communication that is effective for overcoming ambivalence and eliciting motivation for change in a person [33]. MI aims to elicit the desire for behaviour change from the participant; evoking the participants own reasons and capabilities to change promotes commitment to behaviour change [33]. MI has been used successfully for health-related behavioural changes, including in pregnant populations [2, 32] presumably due to the well-established effect MI has shown for initiating change. A previous meta-analysis suggested that MI intervention efficacy is strengthened and prolonged by the incorporation of behaviour maintenance therapies (e.g., CBT) once initial motivation for behaviour change is achieved [34].

CBT refers to a broad range of psychotherapies incorporating cognition therapies with behaviour therapies [35]. It is a facilitated, problem-oriented treatment with a strong evidence base that aims to assist people to understand potentially problematic cognitions and behaviours and facilitate development of more adaptive cognitions and behaviours [35]. CBT has been successful for obesity and weight management, with common techniques such as self-monitoring, goal setting, problem solving and relapse prevention enabling individualised treatment for weight control [36]. Given that cognitions and attitudes can influence GWG [28], CBT techniques can be identified in successful GWG interventions [32, 37] and are effective for sustained behaviour change [38]. Counter to MI, CBT has demonstrated less efficacy in resolving ambivalence to change and is effective for behaviour maintenance when working with individuals that are motivated, incorporating relapse prevention techniques to sustain behaviour change [38, 39].

Traditional interventions prescribing diet and activity changes may not acknowledge or accommodate individual barriers to behaviour change that influence GWG, such as sociocultural, psychological or even physical limitations that accompany pregnancy [3, 40]. Behaviour change interventions that incorporate MI and/or CBT techniques have the potential allow participants to highlight and address barriers to change and support sustained behaviour change [38, 41–43]. Recent meta-analyses confirm that integrated MI-CBT interventions are effective in initiating and maintaining behaviour change to moderate body mass in community dwelling adults and hospital outpatients [44, 45]. However, the provision of MI and CBT techniques to reduce overall weight gain in pregnancy and the likelihood of GWG above or below guidelines has not been systematically evaluated. Therefore, the aim of this study was to determine the effect of interventions incorporating MI and/or CBT techniques on GWG outcomes.

## Methods

### Search strategy

This systematic review and meta-analysis was designed and reported in accordance with guidelines outlined in the Preferred Reporting Items for Systematic Reviews and Meta-Analyses (PRISMA) statement [46]. The protocol for this review was registered with the PROSPERO International register of systematic reviews (registration number CRD42020156401).

A research librarian assisted with developing and undertaking the search strategy. Five electronic databases (CINAHL, Cochrane, PsycINFO, MEDLINE, EMBASE) were searched from inception to March 2022. The search terms were grouped into three concepts: pregnant women, psychosocial intervention and gestational weight change and entered as MeSH terms or keyword combinations and searched with the ‘OR’ operator; resulting search constructs were combined using the ‘AND’ operator (Additional Table S1). An example search strings for CINAHL is attached (Additional Table S2). We also screened reference lists of included papers for potentially relevant studies.

### Screening and eligibility criteria

Following the literature search and removal of duplicates, four reviewers participated in a two-stage screening process, firstly for title and abstract screening, and then full-text articles. A unanimous decision was required from two reviewers to exclude any study, and disagreements were resolved by a third reviewer.

Only original randomised controlled trials published in English language scholarly journals were considered eligible, thus grey literature was excluded. We included interventions examining the effects of MI and/or CBT techniques on GWG or adherence to GWG recommendations. Acknowledging that some interventions may not explicitly identify as MI or CBT, we considered an intervention as MI if it was a facilitated, collaborative therapy whereby initial motivation to change was elicited from the patient, delivered via any mode and not restricted by number or duration of encounters, or professional background of the facilitator [33]. CBT interventions were considered those with a facilitated therapy via human or electronic form with one or more recognised techniques of CBT (both cognitive and behavioural techniques). Intervention descriptions in full texts, supplementary documents and where available protocol documents were examined for the use of MI and CBT processes, relational components and micro-skills. We did not restrict by number or duration of encounters. Criteria for inclusion as a MI or CBT intervention is shown in Additional Table S3. The comparator was standard antenatal care. Excluded studies were those that involved adolescent pregnancy, multiple pregnancy or health conditions that could impact on gestational weight change, and those without a study design measuring gestational weight change.

### Data extraction and analysis

Data describing authors, year of publication, country, participant characteristics, sample size, intervention characteristics, and primary and secondary outcomes were extracted from the included studies into an Excel spreadsheet and exported to Stata for analyses [47]. This process was conducted by one member of the research team, and a second researcher confirmed accuracy.

Meta-analyses were conducted to assess the effect of the intervention on two outcomes: (1) total GWG and (2) adherence to GWG recommendations. To estimate the overall pooled effect of interventions on adherence to GWG guidelines and total GWG, pooled proportions of GWG within and outside guidelines were calculated, with 95% confidence intervals. The effect size for total GWG was estimated using random effects DerSimonian-Laird pooled standardised mean difference (SMD) [48, 49], where SMD of 0.2 represents a small effect, 0.5 a moderate effect and 0.8 a large effect [50]. Heterogeneity between studies was assessed using the I^2^ statistic, with values below 25% representing low heterogeneity, 25% -75% indicating moderate heterogeneity, and values above 75% indicating high heterogeneity [51]. Meta-regressions were performed to measure the proportion of between-study variance explained by covariates including age, risk of bias assessment, BMI, analysis type (intention-to-treat or per protocol), intervention mode of delivery, and weight measurement method. Funnel plots were constructed, and Egger’s test was run to assess for publication bias. All analyses were carried out using Stata/SE 16.0 [47].

### Assessment of Risk of Bias in included studies

The Cochrane Collaboration Risk of Bias 2 tool was used to evaluate the risk of bias for GWG outcomes in included studies across five domains (risk of bias arising from the randomisation process, risk of bias due to deviations from intended interventions, missing outcome data, risk of bias in measurement of the outcome, and risk of bias in selection of the reported result) [52]. According to predetermined criteria set by the Cochrane Collaboration, if a study was assessed as ‘low risk’ across all domains, a score of overall low risk of bias was assigned. If any of the domains were assessed as ‘unclear’ risk of bias, then studies were assigned as unclear risk of bias. A high risk of overall bias resulted if any of the domains were evaluated as high risk of bias. One researcher assessed all included studies for risk of bias, and a second researcher independently evaluated half of the studies to confirm consistency. All studies were included in initial statistical analyses, however studies identified as being ‘high risk’ were removed during sensitivity analyses.

### Quality of evidence

Strength and certainty of the overall evidence was assessed by the research team using the Grading of Recommendations, Assessment, Development and Evaluation [GRADE] system using GRADEpro GDT (GRADEpro Guideline Development Tool [Software]; McMaster University and Evidence Prime, USA). Quality of evidence for meta-analyses began at the high level and were downgraded to lower levels of evidence when risk of bias, inconsistency, indirectness, imprecision or publication bias were deemed to be present. Statements in the results and discussions were presented according to suggested GRADE statements for communicating certainty of evidence [53].

## Results

### Study selection

**Fig. 1:**
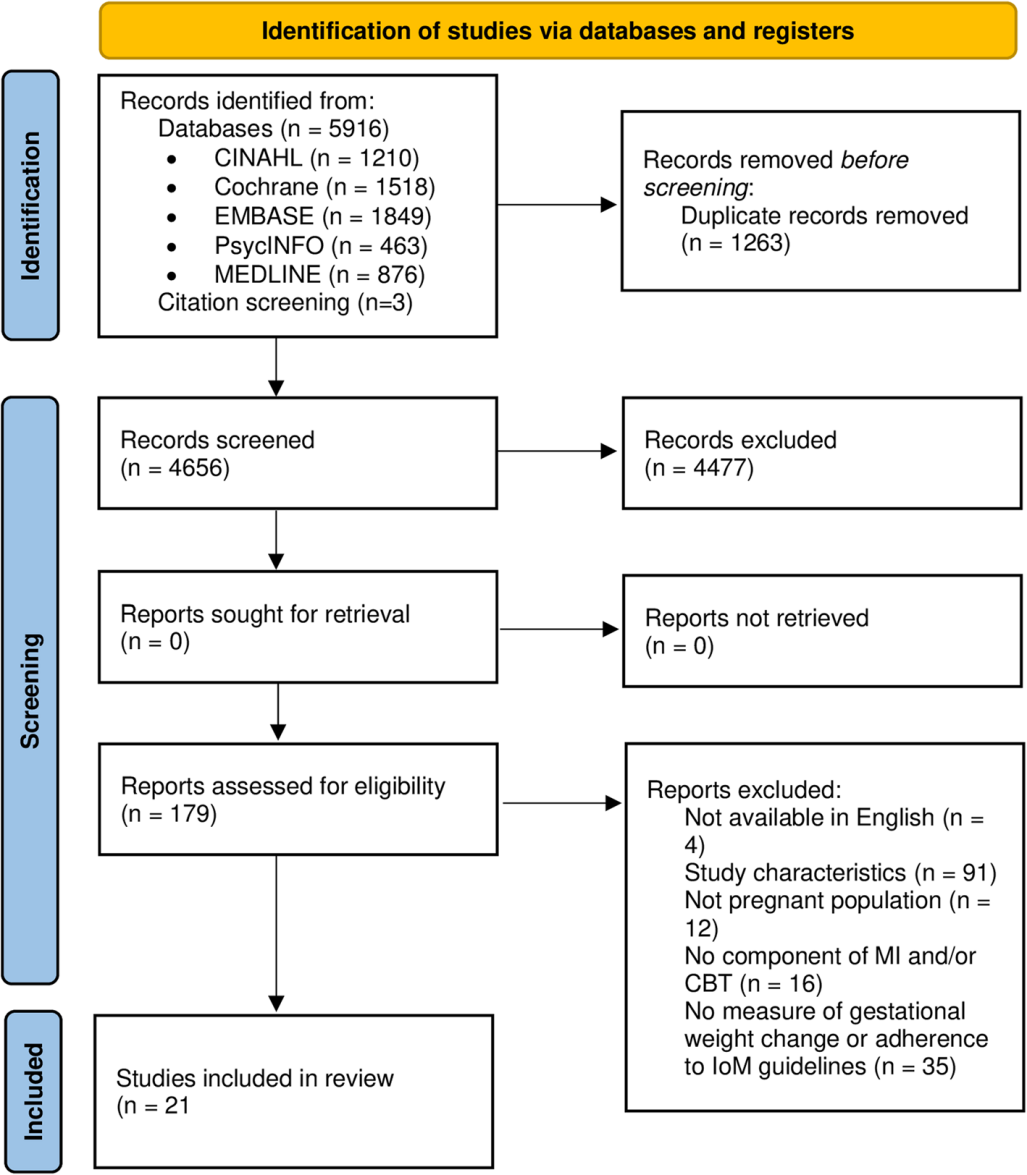
PRISMA flowchart

The search identified 5916 studies, plus three additional studies through citation searching, of which 4656 remained after removing duplicate articles. Of these, 4477 title and abstract records did not meet inclusion criteria and were excluded from further review. 179 full-text articles were assessed for eligibility and a total of 21 studies met criteria for inclusion (Fig. 1). No changes from the registered protocol were required.

Characteristics of the 21 studies (8030 women) included in this systematic review are presented in Table 2. The majority (*n* = 12) of the interventions were conducted in the USA [54–65], four in Europe [66–69], two in Australia [70, 71] and the UK [72, 73], and one in Turkey [74]. Nine of the trials were considered to incorporate CBT techniques only [55, 58–61, 65, 68, 69, 74], 10 trials incorporated a mix of both MI and CBT techniques [56, 62–64, 66, 67, 70–73], and two interventions used MI techniques only [54, 57]. There was variation in the definition of how GWG was calculated; eight studies measured GWG as pre-pregnancy weight deducted from the final weight in pregnancy or at birth [57, 58, 60, 64–66, 69, 73], four studies calculated GWG as pre-pregnancy weight to the final trimester weight before 37 weeks [54, 59, 61, 72], another three measured GWG from a baseline measurement to weight measurement in the final trimester [62, 67, 68]. Furthermore, three studies measured GWG as weight in the first trimester to weight measurement in the final trimester, including on the day of birth [55, 56, 63], one study used weight change from baseline to 28 weeks of pregnancy [70], one study calculated the adjusted total GWG by using a weekly rate of weight gain and multiplying by 40 (for the number of weeks of pregnancy) [71], and one study did not outline how GWG was calculated [74].

**Table 2:** Characteristics of included studies

Study and country	Aim	Sample size	Intervention description	Control^i^	Therapy type – MI/CBT	Pre-pregnancy BMI category	Risk of Bias	Outcomes^a^	Measurement of GWG^b^	Type of weight measurement	Key Findings^c^
**Amanak et al. 2019** [74] Turkey	Evaluate impact of intervention on gestational hypertension and maternal/neonatal outcomes	150	Education booklet based on Roy Adaptation Model plus 2 additional educational meetings	Usual care	CBT	BMI 19–30 kg/m^2^	High	1	❼	Not stated	A
**Bogaerts et al. 2013**[66] Belgium	Evaluate effect of intervention on GWG and anxiety/depression	205	Lifestyle arm: Four small group lifestyle sessions facilitated by a midwife	Usual care	MI/CBT	BMI ≥ 29 kg/m^2^	Unclear	1, 2	❷	Objective measurement	A, B
**Buckingham-Schutt et al. 2019** [54] USA	Evaluate if intervention increased adherence to weight gain guidelines	56	Monthly visits and weekly contact with a registered dietician nutritionist to discuss lifestyle strategies for achieving appropriate GWG	Usual care	MI	BMI 18.5–45 kg/m^2^	Unclear	1, 2	❶	Objective measurement	A, B
**Daley et al. 2019** [73] UK	Assess the effect of a brief behavioural intervention on preventing excessive GWG	656	Intervention involving routine weighing and feedback incorporated into standard antenatal appointments and informed by self-regulation theory	Usual care	MI/CBT	BMI ≥ 18.5	Low	1, 2	❷	Objective measurement	C
**Ferrara et al. 2020** [64] USA	Reduce excessive GWG through a behavioural lifestyle intervention	398	13 weekly sessions targeting behaviour changes for weight management	Usual care	MI/CBT	BMI 25—40	Low	1, 2	❷	Objective measurement	A, B
**Harden et al. 2014** [55] USA	Evaluate impact of intervention on GWG management	16	6 monthly group sessions focusing on behaviour change strategies for diet and exercise	Usual care	CBT	BMI ≥ 30	Unclear	1	❹	Extracted from medical record	A, B
**Harrison et al. 2013** [70] AUS	Optimise GWG and increase adherence to weight gain guidelines	228	4 individual sessions with a health coach to optimise lifestyle and GWG	Enhanced Usual Care (Education session and brochure on diet and exercise guidelines)	MI/CBT	BMI ≥ 25 kg/m^2^	Unclear	1	❺	Objective measurement	A
**Herring et al. 2016** [56] USA	Evaluate if intervention decreases incidence of excessive GWG	66	Technology-based behavioural intervention focusing on energy intake, physical activity and self-weighing with regular health coach calls	Usual care	MI/CBT	BMI 25–45 kg/m^2^	Unclear	1, 2	❹	Extracted from medical record	A, B
**Jackson et al. 2011** [57] USA	Evaluate if intervention is effective for improving pregnant women’s diet and exercise behaviours and incidence of excessive GWG	316	Two sessions with a simulated Video Doctor incorporating nutritional, exercise and weight gain content	Usual care	MI	All	Unclear	1, 2	❷	Extracted from medical record	C
**Kennelly et al. 2018** [68] Ireland	Evaluate effect of intervention on incidence of gestational diabetes mellitus (primary) and GWG (secondary)	565	Education session in conjunction with smartphone app, emails and 2 follow-up sessions all incorporating nutrition, exercise and motivational components	Usual care	CBT	BMI 25–39.9 kg/m^2^	Low	1, 2	❸	Extracted from medical record	A, B
**Liu et al. 2021** [65] USA	Evaluate the effect of intervention on total GWG	228	In-depth counselling session followed by weekly phone counselling and podcasts to promote health behaviour change	Usual care	CBT	BMI ≥ 25 kg/m^2^	Low	1, 2	❷	Extracted from medical record	D
**Olson et al. 2018** [63] USA	Evaluate effect of intervention on incidence of excessive GWG	1689	Online/phone behaviour change intervention with weight tracker, diet and exercise goal setting as well as a variety of health information resources	Enhanced Usual Care (Online/phone access to health information resources only)	MI/CBT	BMI 18.5–35 kg/m^2^	Low	1, 2	❹	Extracted from medical record	C
**Phelan et al. 2011** [58] USA	Evaluate effect of intervention on incidence of excessive GWG	401	Face-to-face then phone lifestyle sessions supported by weekly ‘postcards’	Usual Care	CBT	BMI 19.8–40 kg/m^2^	Unclear	1, 2 recommended GWG	❷	Objective measurement	D
**Phelan et al. 2018** [59] USA	Evaluate effect of intervention on rate of GWG (primary) and incidence of excessive GWG (secondary)	264	Regular in-person lifestyle counselling sessions plus partial meal replacement	Usual Care	CBT	BMI ≥ 25 kg/m^2^	Unclear	1, 2	❶	Objective measurement	A, B
**Polley et al. 2002** [60] USA	Evaluate effect of intervention on incidence of excessive GWG	120	Stepped behavioural sessions focusing on appropriate weight gain, healthy eating and exercise	Usual care	CBT	≥ 19.8 kg/m^2^	Unclear	1, 2	❷	Objective measurement	D
**Poston et al. 2015** [72] UK	Evaluate effect of intervention on incidence of gestational diabetes mellitus (primary) and GWG (secondary)	1555	8 sessions with health lifestyle trainer	Usual care	MI/CBT	BMI ≥ 30 kg/m^2^	Low	1	❶	Objective measurement	A
**Rauh et al. 2013** [69] Germany	Evaluate effect of intervention on incidence of excessive GWG (primary) and GWG	250	2 individual lifestyle counselling sessions	Usual Care	CBT	BMI ≥ 18.5 kg/m^2^	High	1, 2	❷	Objective measurement	A, B
**Simmons et al. 2017** [67] 9 European countries	Compare effectiveness of 3 interventions with usual care on risk for gestational diabetes mellitus and GWG (primary)	436	3 intervention groups: Healthy Eating (HE), Physical Activity (PA), and Healthy Eating + Physical Activity (HE + PA); all receiving 5 face-to-face sessions with 4 phone sessions	Usual care	MI/CBT	BMI ≥ 29 kg/m^2^	Low	1, 2	❸	Objective measurement	E
**Skouteris et al. 2016** [71] AUS	Evaluate effect of intervention on incidence of excessive GWG	261	Individual health coaching session with 2 additional phone sessions 2 × 2 h educational health coaching group sessions	Enhanced Usual Care (2 × 2-h education group sessions)	MI/CBT	All	Low	1, 2	**❻**	Objective measurement	C
**Smith et al. 2016** [61] USA	Evaluate effect of intervention on incidence of excessive GWG	51	Website-based behavioural intervention	Enhanced Usual Care (Website with standard nutrition and activity advice for pregnancy)	CBT	BMI ≥ 18.5 kg/m^2^	Unclear	1, 2	❶	Objective measurement	C
**Vesco et al. 2014** [62] USA	Evaluate the efficacy of intervention on limiting GWG	118	Individual and group counselling sessions for behaviour change, with diet and activity recommendations	Enhanced Usual Care (one session with dietician)	MI/CBT	BMI ≥ 30 kg/m^2^	Low	1, 2	❸	Objective measurement	A, B

^a^Usual Care = routine antenatal care; Enhanced Usual Care = routine antenatal care plus additional feature

Outcomes: 1 = Total GWG; 2 = GWG in relation to IoM recommendations

bMeasurement of GWG: ❶ = Pre-pregnancy to final trimester weight (before 37/40) ❷ = Pre-pregnancy to final antenatal visit weight (including day of birth) ❸ = Baseline to final trimester weight ❹ = First antenatal weight to final antenatal weight (including day of birth) ❺ = Baseline to 28/40 ❻ = Weekly GWG rate * 40 ❼ = Unclear/not defined

cKey Findings Symbols: A = Intervention reduced GWG; B = Intervention reduced excessive GWG/increased appropriate GWG; C = Intervention had no significant effect on GWG and/or appropriate GWG; D = Intervention had some effect on GWG/appropriate GWG in sub-group analysis only; E = multiple intervention arms; at least one intervention arm reduced GWG/increased appropriate GWG

Intervention mode of delivery varied across the included trials. Eight interventions were delivered in-person [55, 59, 62, 66, 69, 70, 73, 74], while four interventions were delivered remotely (i.e., online or via telephone) [56, 57, 61, 63]. Nine interventions used a combination of in-person and remote delivery [54, 58, 60, 64, 65, 67, 68, 71, 72]. Intervention dose varied across trials. Fourteen studies included at least six face-to-face or remote contacts with participants [54–56, 59–65, 67, 68, 72, 73]; the remaining trials involved between two and five contacts with participants [57, 58, 66, 69–71, 74]. Interventions were delivered by a health coach in five trials [56, 67, 70–72], midwives in two trials [66, 73], dietitians in two trials [54, 64] and a mix of professions, including interventionists or research team members in eight trials [55, 58–60, 62, 65, 69, 74], and delivered by phone/computer application or website in four trials [57, 61, 63, 68].

All trials included standard antenatal care as the control; however, five trials also included an additional feature (such as a healthy eating during pregnancy brochure or education session) in the control arm, which were termed ‘Enhanced Usual Care’ [61–63, 70, 71]. Nine trials recruited women from at least three BMI classes (i.e., BMI ≥ 18.5 kg/m^2^) [54, 57, 58, 60, 61, 63, 69, 71, 73], five targeted a BMI ≥ 30.0 kg/m^2^ only [55, 62, 66, 67, 72], six included BMI ≥ 25 kg/m^2^ [56, 59, 64, 65, 68, 70], and one trial limited BMI to between 18.5 – 29.9 kg/m^2^ only [74].

### Risk of Bias in included studies

**Fig. 2:**
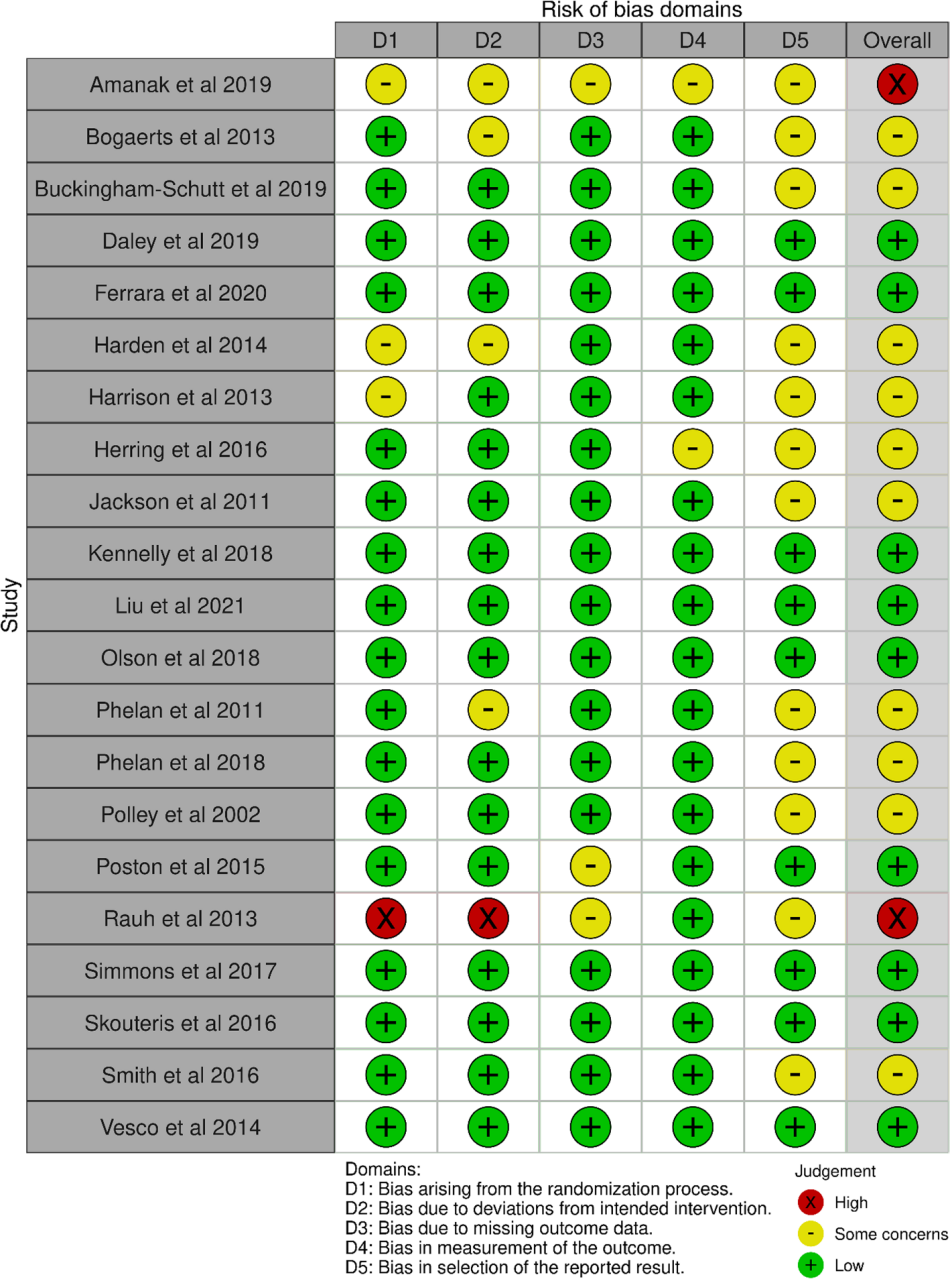
Risk of Bias assessments for included studies

The risk of bias assessment for all studies is presented in Fig. 2. Ten of the studies were rated with overall unclear risk of bias [54–61, 66, 70], nine studies were deemed overall low risk of bias [62–65, 67, 68, 71–73], and two studies were determined to be potentially high risk due to ratings of ‘some concern’ or ‘high’ risk in multiple domains [69, 74].

### GRADE assessment

The overall certainty of evidence for total GWG outcomes was rated as very low due to being downgraded in two risk of bias domains (indirectness and strong likelihood of publication bias). Overall certainty of evidence for adherence to weight gain recommendations was assessed as very low, with the evidence being downgraded due to indirectness and the strong likelihood of publication bias. However, sensitivity analyses to account for high risk of bias produced similar results to the initial meta-analysis. The overall certainty of evidence for the effect of MI and/or CBT techniques on GWG outcomes is presented with further explanation in Additional Table S3.

### Synthesis of results

#### Effect of intervention type on GWG

**Fig. 3:**
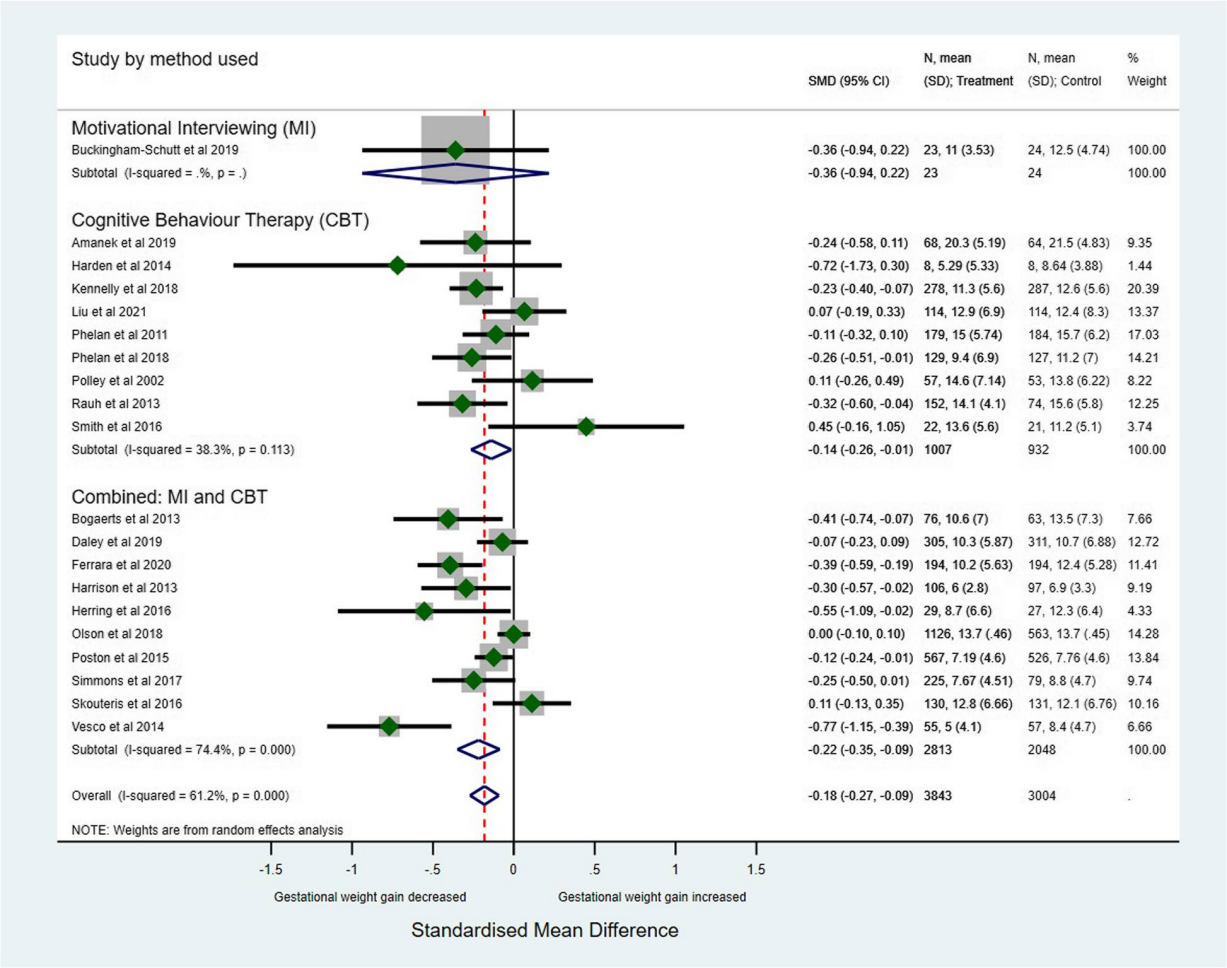
Total Gestational Weight Gain by type of intervention

**Fig. 4:**
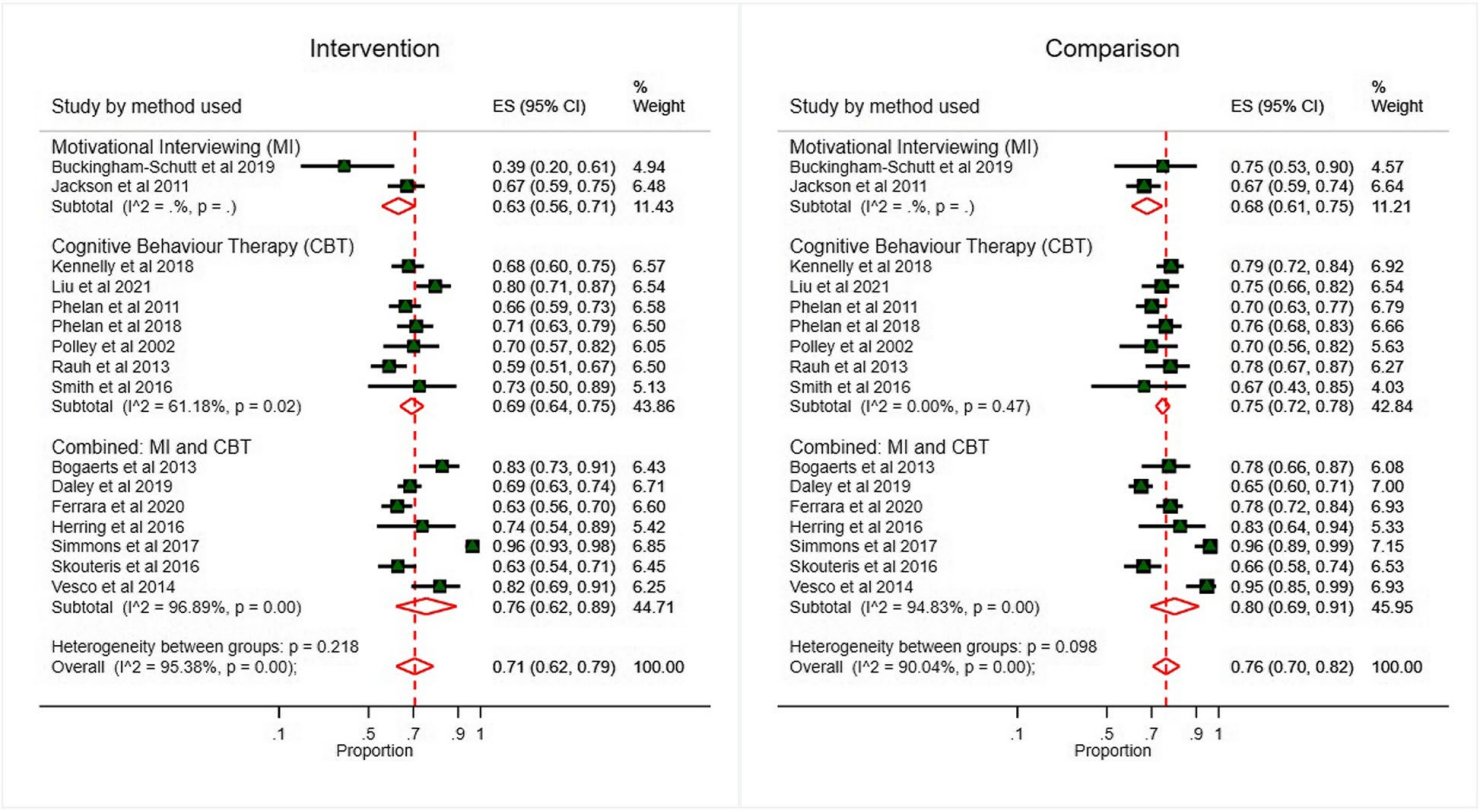
Proportion of women with GWG above or below guidelines stratified by intervention type

The effect of intervention technique (MI alone, CBT alone, and combined MI-CBT interventions) on GWG outcomes is presented in Figs. 3 and 4. There was a small effect for total GWG combined MI-CBT interventions (SMD: -0.22, 95% CI: -0.35 to -0.09, *p* < 0.001) and in CBT alone interventions (SMD: -0.14, 95% CI: -0.26 to -0.01, *p* < 0.001). Combined MI-CBT interventions significantly reduced the proportion of participants with GWG above or below guidelines (76% of intervention participants versus 80% of control participants, *p* = 0.037), as did CBT-alone interventions (69% of intervention participants versus 75% of control participants, *p* = 0.008). MI-alone interventions reduced total GWG (SMD: -0.36, 95% CI: -0.94 to -0.22; one study) and decreased GWG above or below guidelines GWG (63% of intervention participants versus 68% of control participants, *p* = 0.337, two studies), although the data lacked power to show statistical significance.

#### Effect of interventions on total GWG

**Fig. 5:**
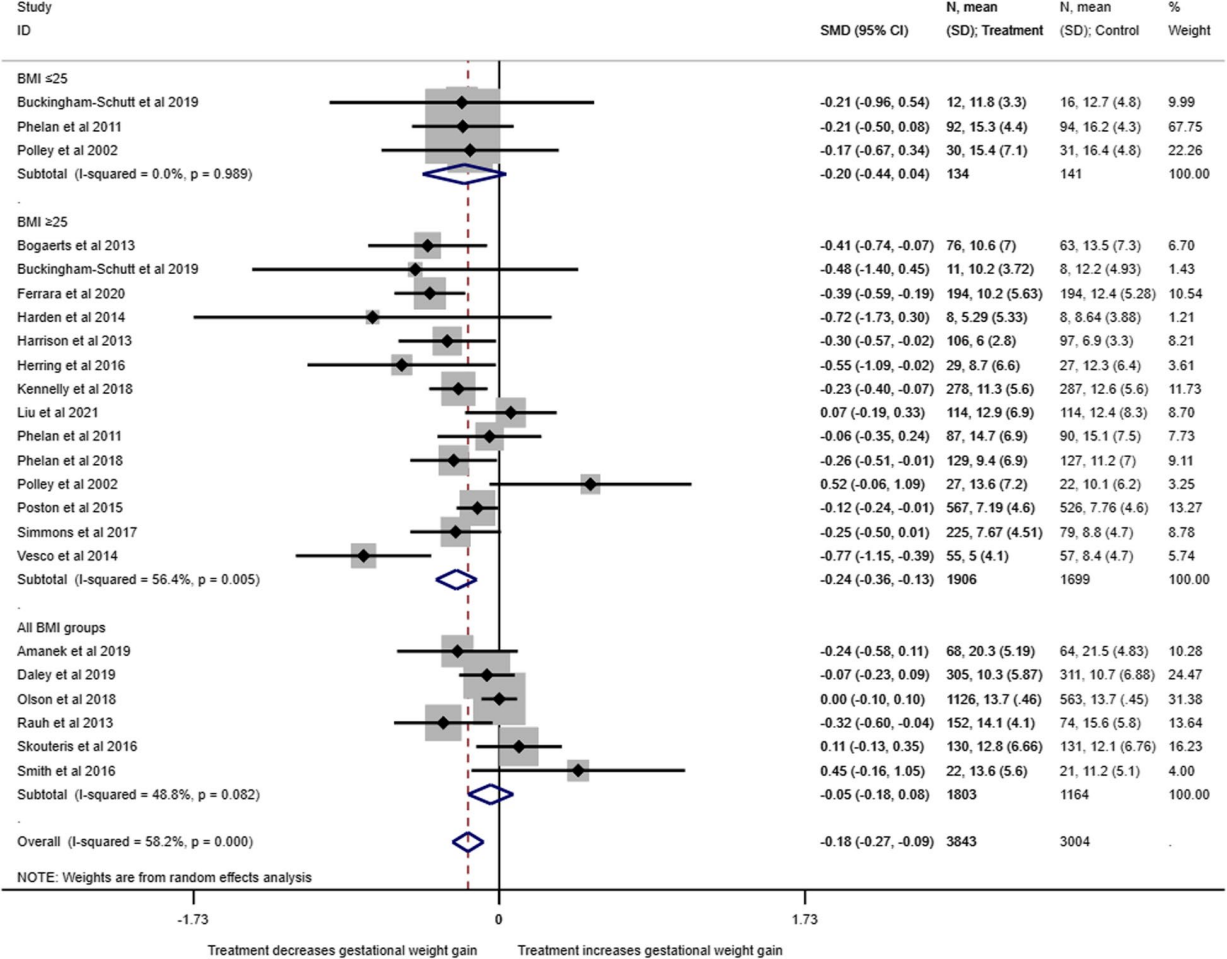
Total Gestational Weight Gain stratified by BMI category

Twenty trials involving 7656 women (95%), evaluated the effect of MI and/or CBT interventions on total weight gain in pregnancy. Overall, compared with women in the control arm, the intervention arm produced a small effect on total GWG (SMD: -0.18, 95% CI: -0.28 to -0.09, *p* < 0.001) (Fig. 5). There was a greater effect on women with a BMI ≥ 25 kg/m^2^ where there was a reduction in weight gain (SMD: -0.24, 95% CI: -0.36 to -0.13, *p* = 0.005) compared with studies that included all BMI classes (SMD: -0.05, 95% CI: -0.18 to 0.08, *p* = 0.082) or BMI < 25 kg/m^2^ (SMD: -0.20, 95% CI: -0.44 to 0.04, *p* = 0.989). Heterogeneity between studies was moderate (I^2^ = 58.2%, p ≤ 0.001). Interventions with MI and/or CBT techniques may reduce total GWG, but the evidence is very uncertain.

#### Effect of interventions on achieving appropriate weight gain

**Fig. 6:**
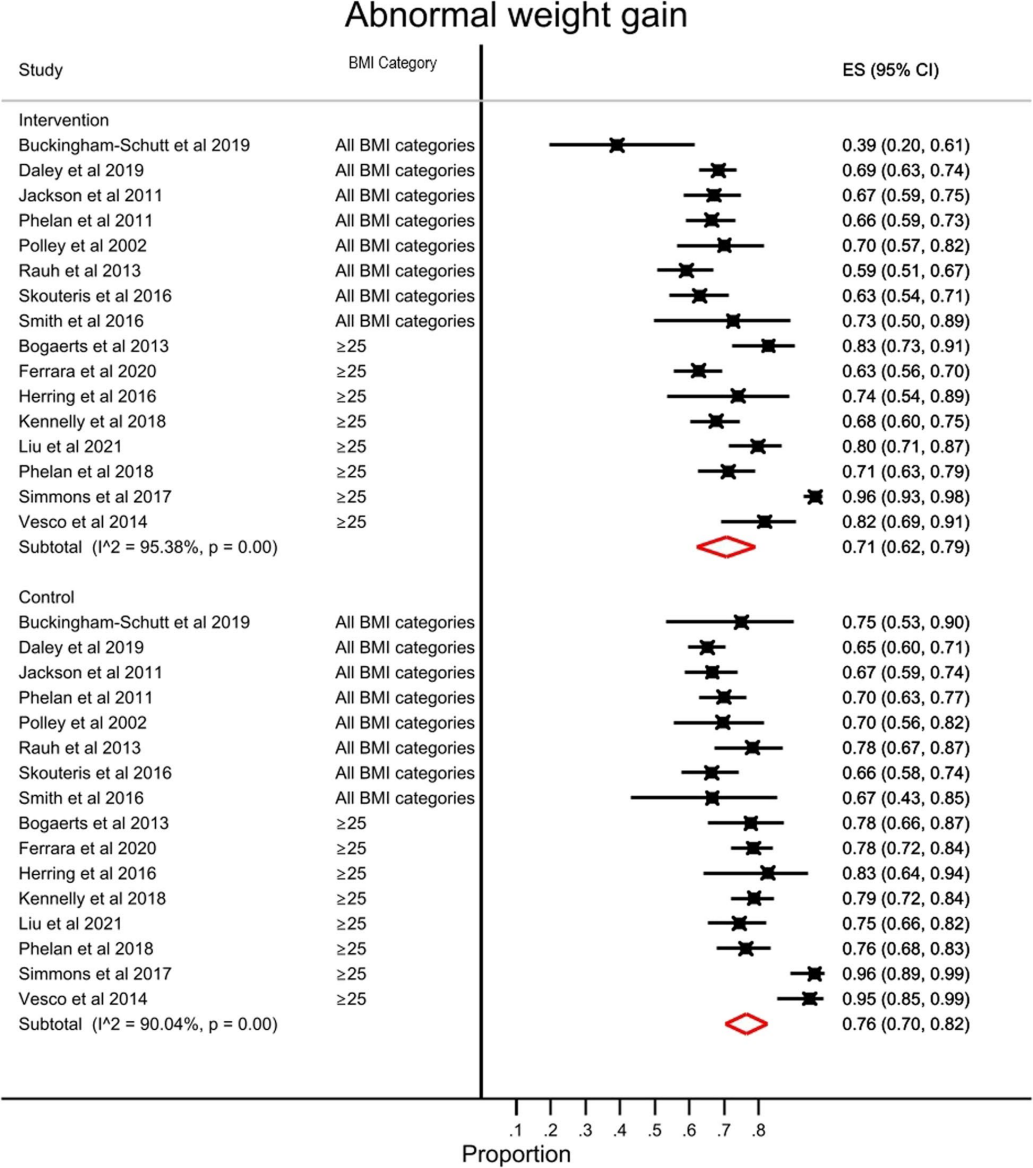
Proportion of women with GWG above or below guidelines

Sixteen (76%) of the included studies (4336 women) evaluated the adequacy of participants’ weight gain against GWG recommendations. Overall, the MI and/or CBT interventions reduced the proportion of women with GWG outside the GWG recommendations (71% of intervention participants versus 77% of control participants, *p* < 0.001) (Fig. 6). Analysis by BMI class showed the intervention significantly reduced the likelihood of GWG above or below guidelines in women with overweight or obesity (76% of intervention participants versus 81% of control participants, *p* = 0.006) and in women with a BMI < 25 kg/m^2^ (54% of intervention participants versus 67% of control participants, *p* = 0.027) (Additional Fig. S1). A similar finding was noted in interventions that included all BMI classes; however, the data lacked power to show statistical significance (66% of intervention participants versus 68% of control participants, *p* = 0.421). Heterogeneity between studies was high (I^2^ = 90.0%, *p* < 0.001). Interventions with components of MI and/or CBT may increase the proportion of women achieving appropriate GWG that is important, but the evidence is very uncertain.

#### Sensitivity analysis and meta-regression

Sensitivity analysis, where meta-analyses were performed after removing studies with high risk of bias, yielded similar results to the original meta-analysis (Additional Fig. S2).

Meta-regressions were performed to assess proportion of between-study variance explained by study characteristics. Only BMI category and intervention mode of delivery were found to contribute significantly to between-study variance, explaining 30.4% and 37.2% of between study variance respectively. Consequently, a sub-group analysis was conducted by intervention mode of delivery, as sub-group analyses by BMI class were performed prior to this. The forest plots were stratified by three different intervention delivery modes (i.e., face-to-face delivery, remote delivery, or a combination of face-to-face and remote delivery) to account for this variable. The intervention successfully limited total GWG in interventions delivered in-person (SMD: -0.31, 95% CI: -0.46 to -0.16) or via a combination of in-person/remote delivery (SMD: -0.13, 95% CI: -0.24 to -0.02), but not in remote delivery only interventions (SMD: -0.04, 95% CI: -0.46 to 0.38) (Additional Fig. S3). The intervention was successful in reducing GWG above or below guidelines when delivered in-person (*p* = 0.003) and in-person/remote combinations (*p* = 0.003), but not when solely delivered remotely when compared to the control condition for the respective mode of delivery (*p* = 0.847) (Additional Fig. S4).

The funnel plots of included studies show asymmetry (*p* < 0.005), indicating possible publication bias where studies with successful interventions were more likely to be published (Additional Fig. S5).

## Discussion

To our knowledge, this is the first systematic review with meta-analyses to investigate the effect of behaviour change interventions using MI and/or CBT techniques on GWG outcomes. Inclusion of MI-CBT techniques appear to be effective for improving the proportion of women with appropriate GWG (29% in the intervention participants, compared to 24% in standard antenatal care), an outcome that is linked with significant short- and long-term health outcomes for mothers and infants when compared with standard antenatal care, although substantial heterogeneity was noted. The effects were greater in women with overweight or obesity. The finding is important given this cohort of women are at risk of GWG above guidelines and associated sequelae. The results of the meta-analyses support the use of CBT or integrated MI-CBT techniques as an approach for achieving appropriate GWG. Due to concerns with risk of bias, indirectness and potential for publication bias, the overall certainty in the evidence is very uncertain. These findings suggest that MI-CBT techniques can affect behaviour change that influences weight and health-related outcomes, however, data were not available to evaluate for sustained behaviour change or longer-term outcomes such as postpartum weight retention, which would present additional benefits to pregnant people from these interventions.

Interventions including CBT-alone, or integrated MI and CBT techniques significantly reduced total GWG and the proportion of participants with GWG outside recommendations. This finding is consistent with existing literature demonstrating significant effects from combined MI-CBT interventions [43–45]. Studies indicate that with each 1 kg reduction in GWG there is an associated impact of reduced risk for LGA, reduced infant birth weight (by 17–23 g) and postpartum weight retention (by -0.88 kg) [3, 75], thus the finding of an overall significant difference with a small effect for total GWG may have a clinical impact in these areas. Interventions delivered in-person, or in-person combined with remote delivery, were also successful in limiting GWG and the proportion of participants with GWG above or below guidelines. Similar effects were seen in MI-alone interventions, and interventions delivered exclusively through remote methods, however lacked statistical power. Conducting further research involving MI-only interventions, and/or exclusively remote-delivery interventions, would strengthen the evidence in relation to the effect of these intervention types on GWG outcomes.

Consistent with previous reviews that focus on pregnant women with overweight or obesity [76, 77], this systematic review found the intervention effect on GWG and proportion of women achieving adequate GWG was greater in women with a BMI > 25 kg/m^2^; reducing the proportion of women with GWG above or below guidelines compared to the control condition (76% versus 81%). These BMI categories are associated with additional risks of adverse perinatal outcomes [76], and are more likely to have GWG above guidelines than other BMI categories [77]. Given the consistently high proportion of pregnant women with overweight or obesity that do not achieve appropriate GWG, further interventions are warranted that include additional considerations aimed at increasing maternal GWG adequacy.

The findings of this review also confirm that a substantial proportion of pregnant women do not achieve target GWG [4, 78]. The findings confirm that GWG above or below guidelines during pregnancy continues to represent a significant issue for maternity care providers and suggests that other factors need consideration when designing interventions for GWG. Our findings support previous evidence that components of MI and CBT techniques are effective for initiating and sustaining behaviour change, in relation to GWG.

Intervention mode of delivery influenced the intervention effect. Only interventions delivered in-person, or remotely in combination with in-person, successfully reduced GWG and significantly decreased the proportion of women with weight gain outside of GWG recommendations when compared to the respective control. This contributes to evidence regarding optimal mode of delivery, which has been inconsistent. A recent review of 12 studies found telephone-based interventions such as telephone calls and short messaging service were effective for GWG control [79]. However, evaluation of other telephone functions such as smartphone applications, social networks and video calls were not evaluated, as studies using these telephone functions were not included [79]. Previous reviews concluded evidence was lacking to support or refute the use of technology-based interventions for GWG [80–82]. Our findings suggest that while in-person intervention delivery is important for pregnant women, telephone and technology follow-up can provide adequate support to this contact and this has the advantage of intervention delivery that is flexible, brief, and cost-effective. This mode of delivery is also highly accessible for a diverse population of pregnant women, such as those living in rural and remote regions, where there are higher rates of obesity [83, 84].

### Limitations and future recommendations

While our meta-analyses suggest there is a positive effect of MI and/or CBT techniques on GWG-related outcomes, a significant proportion of pregnant women do not achieve GWG within recommended ranges. Further research trialling psychosocial interventions to support behaviour change for appropriate GWG is warranted because improved control of GWG will reduce the risk of adverse perinatal outcomes for women and infants.

We excluded non-English language articles, which could mean that some relevant studies were not included. Measurement of GWG varied across studies, and therefore the reported GWG might not reflect the total GWG over the course of the entire pregnancy. Most interventions included in these meta-analyses involved combined MI and CBT, or CBT only techniques. Only two interventions trialled only MI techniques and thus MI-only interventions are less represented in these data. Further research trialling MI techniques on GWG outcomes would strengthen the evidence relating to the effect of MI techniques on GWG-related outcomes. While meta-regressions and sensitivity analysis did not change results, the complex and multi-component nature of the included interventions indicates notable variability exists between interventions where an effect of unknown variables cannot be ruled out.

Although we evaluated GWG both above and below guidelines, many of the included studies focused on limiting excessive GWG or total GWG only, and five of the included studies did not have GWG as a primary outcome measure of their intervention. There was variation across included studies in how GWG was defined and/or measured. A consistent approach to the definition and measurement of GWG is needed in future research. The training provided to intervention facilitators or measured intervention fidelity was not reported in all studies. As a result, the extent to which individuals were receiving MI and CBT techniques is uncertain. Future studies examining interventions based on MI and/or CBT should include a measurement of fidelity into the study design.

## Conclusions

These meta-analyses show that MI and/or CBT techniques can reduce total GWG and improve the proportion of pregnant women with appropriate GWG. However, the collective evidence is very uncertain, and a large proportion of women still do not achieve normal GWG in pregnancy. These findings support existing evidence that MI and/or CBT techniques can be effective in initiating and sustaining behaviour change when delivered in-person or via a combination of remote and in-person delivery. Future research should consider and incorporate additional factors that contribute to maternal GWG, including exploration of clinician and women’s perspectives regarding intervention design and delivery.

## Supplementary Information

**Additional file 1: Table S1.** Example search strategy.

**Additional file 2: Table S2.** Example database search strings for CINAHL database.

**Additional file 3: Table S3.** Intervention features for Motivational Interviewing and Cognitive Behaviour Therapy.

**Additional file 4:  Table S4.** Summary of findings.

**Additional file 5: Figure S1.** Proportion of participants with inappropriate GWG stratified by BMI category.

**Additional file 6: Figure S2.** Sensitivity analysis.

**Additional file 7: Figure S3.** Total GWG stratified by intervention mode of delivery.

**Additional file 8: Figure S4.** Proportion of participants with inappropriate GWG stratified by intervention mode of delivery.

**Additional file 9: Figure S5.** Funnel plot.

## Data Availability

All data and materials used in this research are available via the corresponding author on request. References are provided.

## Abbreviations

BMI: Body mass index
CBT: Cognitive Behaviour Therapy
CI: Confidence interval
GDM: Gestational diabetes mellitus
GWG: Gestational weight gain
LGA: Large for gestational age
MI: Motivational Interviewing
