# Supplementary material for: The effect of motivational interviewing and/or cognitive behaviour therapy techniques on gestational weight gain – a systematic review and meta-analysis
Source: BMC Public Health. 2023 Apr 1;23:626. doi: 10.1186/s12889-023-15446-9 (PMC10067184; doi:10.1186/s12889-023-15446-9)
Supplement: Supplementary file 1 — Additional file 1: Table S1. Example search strategy. [file 12889_2023_15446_MOESM1_ESM.docx]

Additional Table S1 – Example search strategy

| PICOS components | Details |
| --- | --- |
| Population Pregnant women | ‘Pregnant women’ (MeSH) OR ‘Expectant mothers’ OR ‘Pregnancy’ OR ‘Pregnan*’ OR ‘Gravid women’ |
| Intervention  Motivational interviewing or Cognitive Behaviour Therapy | ‘Psychotherapy, Brief’ (MeSH) OR ‘Psychotherapy’ OR ‘Combined psychotherapy*’ OR ‘Motivational interviewing’ (MeSH) OR ‘Motiv* Interv*’ OR ‘Motiv* counsel*’ OR ‘health* coach*’ OR ‘Cognitive Therapy’ (MeSH) ‘Cognitive Therap*’ OR ‘CBT’ OR ‘Cognit* Behav* Therap*’ OR ‘Cognit* Behav* Strat*’ OR ‘CBS’ OR ‘Behav* Therap*’ OR Cognit* Restruct*’ OR ‘Rational Emotive Therap*’ OR ‘RET’ OR ‘Behav* chang’ OR ‘health* behav*’ |
| Outcomes  Gestational weight change | ‘Gestational weight gain’ (MeSH) OR ‘Weight gain’ OR ‘weight control’ OR ‘weight change’ OR ‘weight gain’ OR ‘weight’ OR ‘body mass’ |
