# Supplementary material for: The effect of motivational interviewing and/or cognitive behaviour therapy techniques on gestational weight gain – a systematic review and meta-analysis
Source: BMC Public Health. 2023 Apr 1;23:626. doi: 10.1186/s12889-023-15446-9 (PMC10067184; doi:10.1186/s12889-023-15446-9)
Supplement: Supplementary file 2 — Additional file 2: Table S2. Example database search strings for CINAHL database. [file 12889_2023_15446_MOESM2_ESM.docx]

Additional Table S2 – Example database search strings for CINAHL database

| **Database** | **Search strings** | **`Search option** | **Outcomes** |
| --- | --- | --- | --- |
| CINAHL | (MH “Pregnancy+) OR (MH “Expectant Mothers”) OR (pregnan*) OR (“expectant mother”) OR (“gravid women”) AND (MH Psychotherapy, Brief”) OR (“Combined psychotherapy*”) OR (MH “Motivational Interviewing”) OR (“motiv* interv*”) OR (“motiv* counsel*) OR (“health* coach*”) OR (“motiv* N2 change”) OR (MH “Cognitive Therapy+”) OR (“Cognitive N2 Therap*”) OR (“CBT”) OR (“Cognit* Behav* Therap*”) OR (“Cognit* Behav* Strat*”) OR (“CBS”) OR (“Behav* Therap*”) OR (“Cognit* Restructur*”) OR (“Rational Emotive Therap*”) OR (“RET”) OR (MH “Behavioural Changes”) OR (“behav8 chang*”) OR (“health* behav*”) AND (MH “Gestational weight gain”) OR (MH “Weight Gain+”) OR (MH “Weight Control”) OR (MH “Body Weight Changes+”) OR (“weight gain”) OR (“weight”) OR (“body mass”) | Search mode – Boolean/Phrase; Limiters – English Language; Huma; | 1210 |
