# Supplementary material for: The effect of motivational interviewing and/or cognitive behaviour therapy techniques on gestational weight gain – a systematic review and meta-analysis
Source: BMC Public Health. 2023 Apr 1;23:626. doi: 10.1186/s12889-023-15446-9 (PMC10067184; doi:10.1186/s12889-023-15446-9)
Supplement: Supplementary file 3 — Additional file 3: Table S3. Intervention features for Motivational Interviewing and Cognitive Behaviour Therapy. [file 12889_2023_15446_MOESM3_ESM.docx]

Additional Table S3 – intervention features for Motivational Interviewing and Cognitive Behaviour Therapy

| Intervention | Intervention features |
| --- | --- |
| Motivational Interviewing | A facilitated, guided communication technique where ambivalence to change is overcome, and motivation to change is elicited from the patient, using accepted MI techniques (such as engaging, focusing or planning techniques).  Delivered through any mode (e.g., counsellor, nurse, electronic).  Not restricted by number or duration of encounters. |
| Cognitive Behaviour Therapy | A facilitated therapy, either via human or electronic form.  Includes both cognition therapies (such as cognitive restructuring, goal setting, problem solving) and behaviour therapies (resolution strategies, relapse prevention, self-monitoring), or strategies with combined cognitive and behaviour change targets.  Not restricted by number or duration of encounters |
