# Supplementary material for: The effect of motivational interviewing and/or cognitive behaviour therapy techniques on gestational weight gain – a systematic review and meta-analysis
Source: BMC Public Health. 2023 Apr 1;23:626. doi: 10.1186/s12889-023-15446-9 (PMC10067184; doi:10.1186/s12889-023-15446-9)
Supplement: Supplementary file 4 — Additional file 4: Table S4. Summary of findings. [file 12889_2023_15446_MOESM4_ESM.docx]

Additional Table S4 – summary of findings

| **Certainty assessment** | | | | | | | **№ of patients** | | **Effect** | | **Certainty** | **Importance** |
| --- | --- | --- | --- | --- | --- | --- | --- | --- | --- | --- | --- | --- |
| **№ of studies** | **Study design** | **Risk of bias** | **Inconsistency** | **Indirectness** | **Imprecision** | **Other considerations** | **motivational interviewing and/or cognitive behaviour therapy** | **usual care** | **Relative (95% CI)** | **Absolute (95% CI)** |  |  |
| **Total GWG** | | | | | | | | | | | | |
| 20 | randomised trials | serious^a^ | not serious | serious^b^ | not serious | publication bias strongly suspected^c^ | 3843 | 3004 | - | SMD **0.18 SD lower** (0.27 lower to 0.09 lower) | ⨁◯◯◯ Very low | IMPORTANT |
| **Adherence to weight gain recommendations** | | | | | | | | | | | | |
| 16 | randomised trials | serious^a^ | not serious | serious^b^ | not serious | publication bias strongly suspected^c^ | 1424/1998 (71.3%) | 1343/1802 (74.5%) | **RR 0.922** (0.781 to 1.087) | **6 fewer per 100** (from 16 fewer to 6 more) | ⨁◯◯◯ Very low | IMPORTANT |

**CI:** confidence interval; **RR:** risk ratio; **SMD:** standardised mean difference

#### Explanations

a. Many included studies rated high or unclear risk of bias

b. Differences in population, sub-groups, outcome measures, interventions

c. asymmetry in pattern of results
