# Supplementary material for: The effect of motivational interviewing and/or cognitive behaviour therapy techniques on gestational weight gain – a systematic review and meta-analysis
Source: BMC Public Health. 2023 Apr 1;23:626. doi: 10.1186/s12889-023-15446-9 (PMC10067184; doi:10.1186/s12889-023-15446-9)
Supplement: Supplementary file 5 — Additional file 5: Figure S1. Proportion of participants with inappropriate GWG stratified by BMI category. [file 12889_2023_15446_MOESM5_ESM.docx]

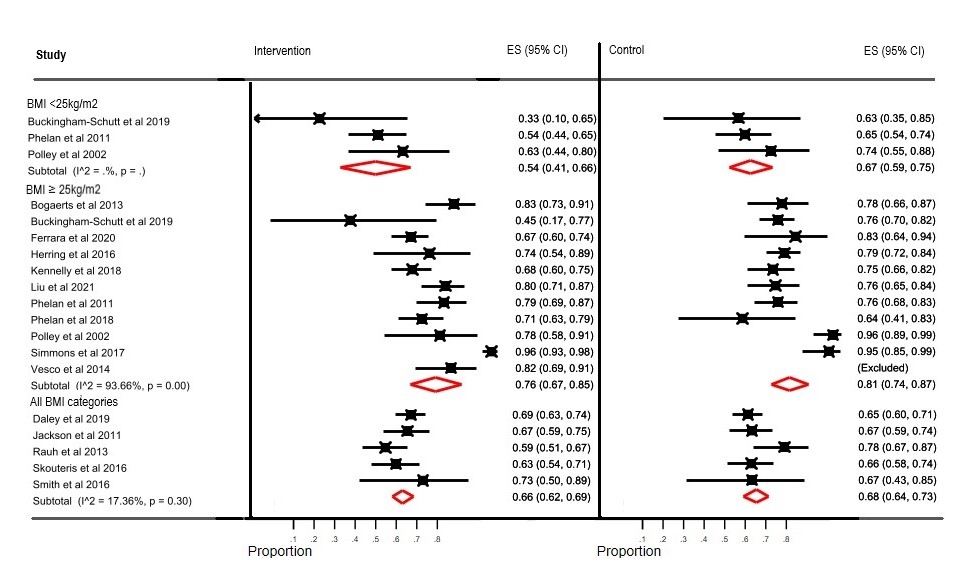


Additional figure S1 – proportion of participants with inappropriate GWG stratified by BMI category
