# Supplementary figures and images for: The effect of motivational interviewing and/or cognitive behaviour therapy techniques on gestational weight gain – a systematic review and meta-analysis
Source: BMC Public Health. 2023 Apr 1;23:626. doi: 10.1186/s12889-023-15446-9 (PMC10067184; doi:10.1186/s12889-023-15446-9)

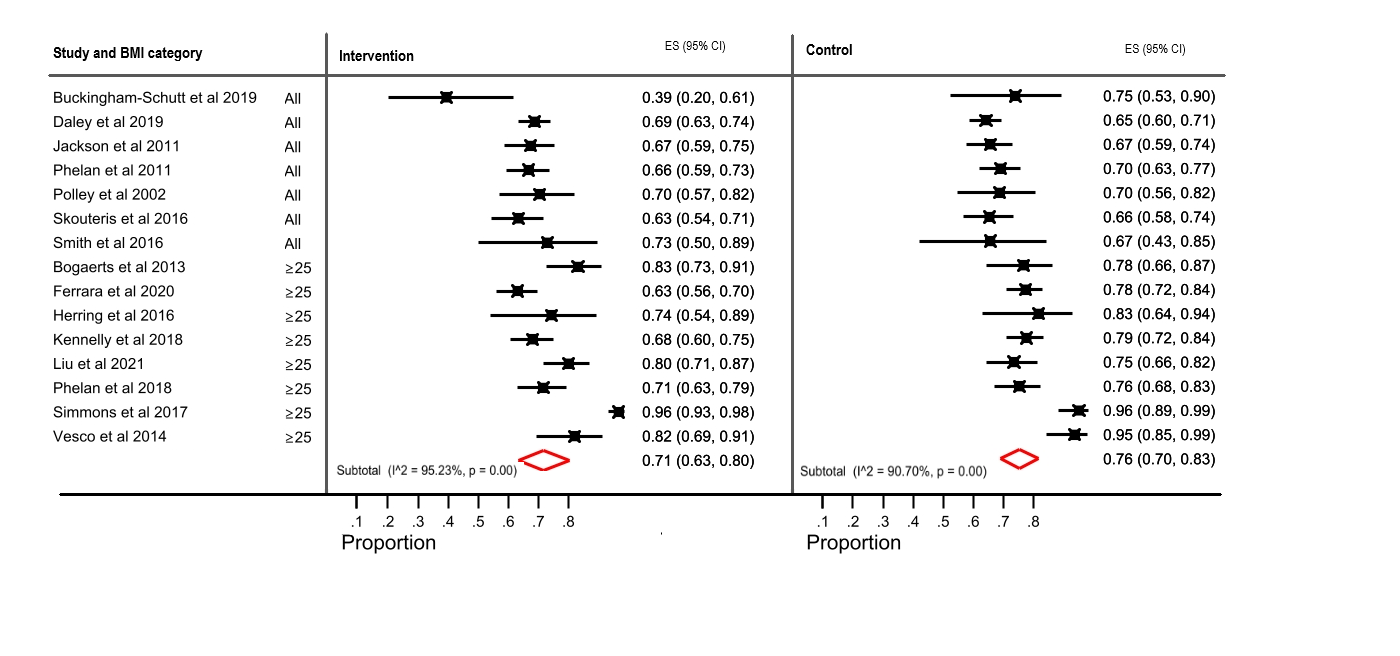


Additional figure S2 – sensitivity analysis

Supplement: Supplementary file 6 — Additional file 6: Figure S2. Sensitivity analysis. [file 12889_2023_15446_MOESM6_ESM.docx]

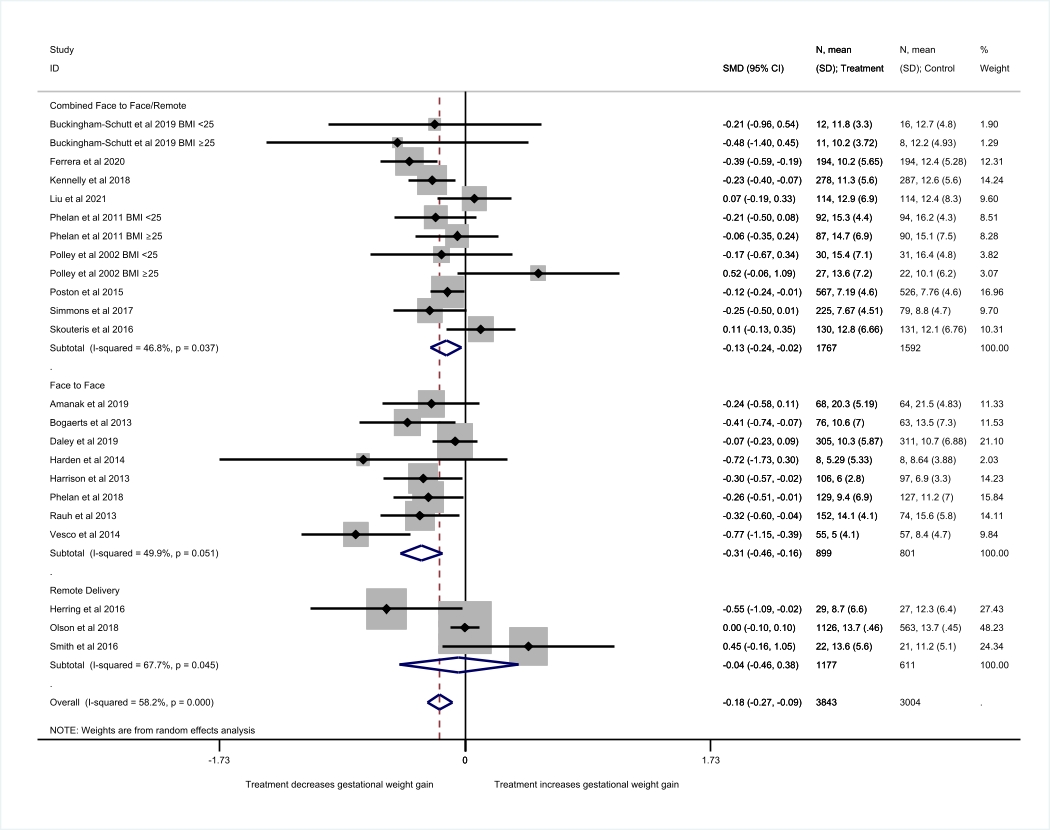


*Additional figure S3 – Total GWG stratified by intervention mode of delivery*

Supplement: Supplementary file 7 — Additional file 7: Figure S3. Total GWG stratified by intervention mode of delivery. [file 12889_2023_15446_MOESM7_ESM.docx]

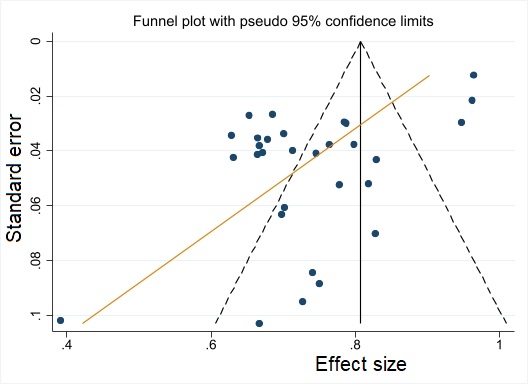


Additional figure S5 – Funnel plot

Supplement: Supplementary file 9 — Additional file 9: Figure S5. Funnel plot. [file 12889_2023_15446_MOESM9_ESM.docx]
